# Ectopic adrenal tissue around the prostate: A rare case

**DOI:** 10.1097/MD.0000000000046287

**Published:** 2026-05-12

**Authors:** Jiagui Chai, Runlin Feng, Anlian Wang, Tao Zhang, Yuhang Li, Yingxiong Yang, Guorun Zi, Changxing Ke

**Affiliations:** a Department of Urology, Mengzi People’s Hospital Affiliated to Yunnan University of Traditional Chinese Medicine, Mengzi, China; b Department of Urology, The Second Affiliated Hospital of Kunming Medical University, Kunming, China; c Department of Pathology, The Second Affiliated Hospital of Kunming Medical University, Kunming, China; d Department of Urology, Yunnan Northeastern Central Hospital, Zhaotong, China.

**Keywords:** case report, clinical features, ectopic adrenal tissue, origin, prostate

## Abstract

**Rationale:**

Adrenal ectopia is defined as the presence of adrenal tissue in a location other than the adrenal glands. Ectopic adrenal tissue has been reported in the region around the adrenal gland, the celiac plexus, the kidney, and the route of gonadal descent, comprising also hernia sacs. However, to our knowledge, ectopic adrenal tissue around the prostate is extremely rarely reported.

**Patient concern:**

A 56-year-old male was admitted to the hospital due to 4 days of gross hematuria.

**Diagnoses:**

Magnetic resonance imaging showed multiple lesions on the bladder wall, with the larger one located on the posterior wall of the bladder and partially invading the muscle. We combined with preoperative imaging and diagnosed a muscle-invasive bladder cancer.

**Interventions:**

The patient underwent radical cystoprostatectomy.

**Outcomes:**

The surgery was successfully performed. Postoperative pathology indicated invasive papillary urothelial carcinoma. Unexpectedly, postoperative pathology also revealed the presence of adrenal tissues around the prostate (The adrenal tissue was located outside the prostatic capsule). The diagnosis of ectopic adrenal tissue around the prostate was considered. The patient was discharged 9 days after surgery and no significant abnormalities were found during follow-up.

**Lessons:**

This case represents the ectopic adrenal tissue around the prostate, broadening our understanding of adrenal tissue ectopia. Ectopic adrenal tissue around the prostate is usually discovered by chance. It contains only the cortex and appears to be nonfunctional. Due to its small size and rarity, detecting ectopic adrenal tissue in the prostate through imaging is challenging.

## 1. Introduction

The adrenal glands are retroperitoneal organs typically situated above the kidneys. However, adrenal tissue can become ectopic due to congenital embryonic developmental abnormalities.^[1]^ The earliest recorded instance of ectopic adrenal tissue dates back to the 18th century when adrenocortical tissue was discovered in the spermatic cord of a child.^[2]^ Since then, ectopic adrenal tissue has been identified in various locations, including the kidney, hernia sac, and placenta.^[3]^ However, to our knowledge, ectopic adrenal tissue around the prostate is extremely rarely reported. In this article, we describe a rare case of ectopic adrenal tissue around the prostate, aiming to provide new insights of the disease.

## 2. Case presentation

**Figure 1.**
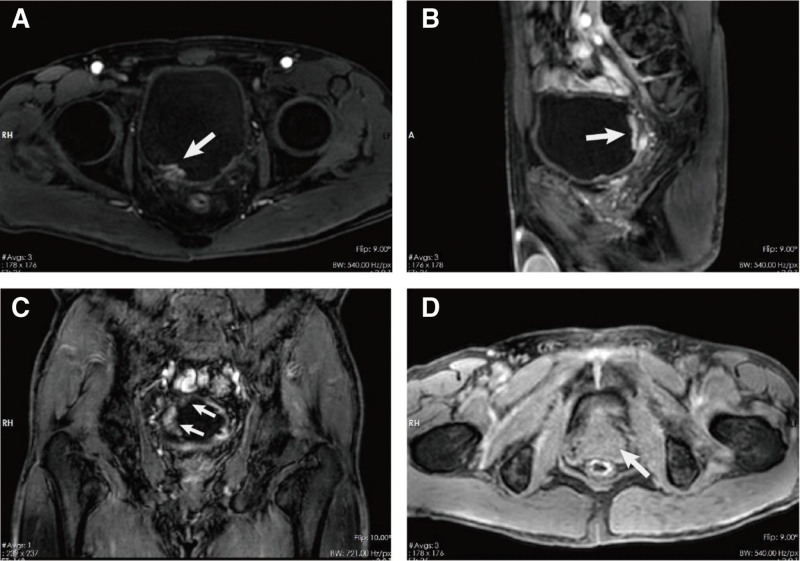
MRI of the patient before surgery. MRI showed multiple lesions on the bladder wall in the transverse (A), sagittal (B), and coronal planes (C), with the larger one located on the posterior wall of the bladder, measuring approximately 1.6 × 1.0 × 0.55 cm. (D) No abnormalities were detected in the prostate. MRI = magnetic resonance imaging.

A 56-year-old male was admitted to the hospital due to 4 days of gross hematuria. He had a medical history of hypertension and diabetes for 4 years. Hypertension and diabetes are well treated with medication. The physical examination was unremarkable. Urine analysis indicated an elevated urine red blood cell count. magnetic resonance imaging showed multiple lesions on the bladder wall, with the larger one located on the posterior wall of the bladder and partially invading the muscle, measuring approximately 1.6 × 1.0 × 0.55 cm. Enhanced magnetic resonance imaging scanning demonstrated significant enhancement of the lesions. No abnormalities were detected in the peribladder fat, prostate, bilateral seminal vesicles, rectal wall, pelvic lymph nodes, and inguinal area (Fig. 1A–D).

We combined with preoperative imaging and diagnosed a muscle-invasive bladder tumor.

## 3. Treatment

Surgery is recommended. Radical cystoprostatectomy was performed. Postoperative pathology indicated invasive papillary urothelial carcinoma.

**Figure 2.**
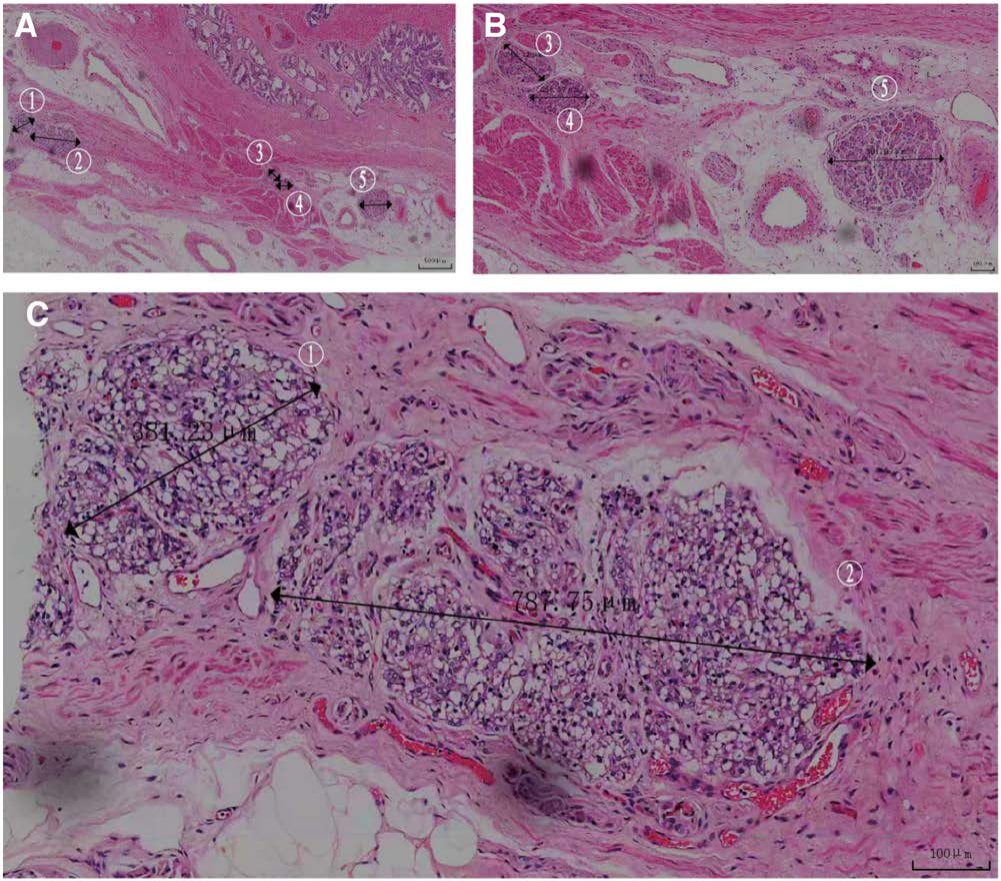
A diagram of the pathology. (A) Adrenal tissue is found around the prostate with 5 lesions (HE staining, scale length: 500 μm). (B) Adrenal tissue is composed of adrenal cortex and arranged in a nest-like distribution (HE staining, scale length: 100 μm). (C) The cell and nucleus are large, and the cytoplasm contains a large number of lipid droplets in a foamy shape (HE staining, scale length: 100 μm).

**Figure 3.**
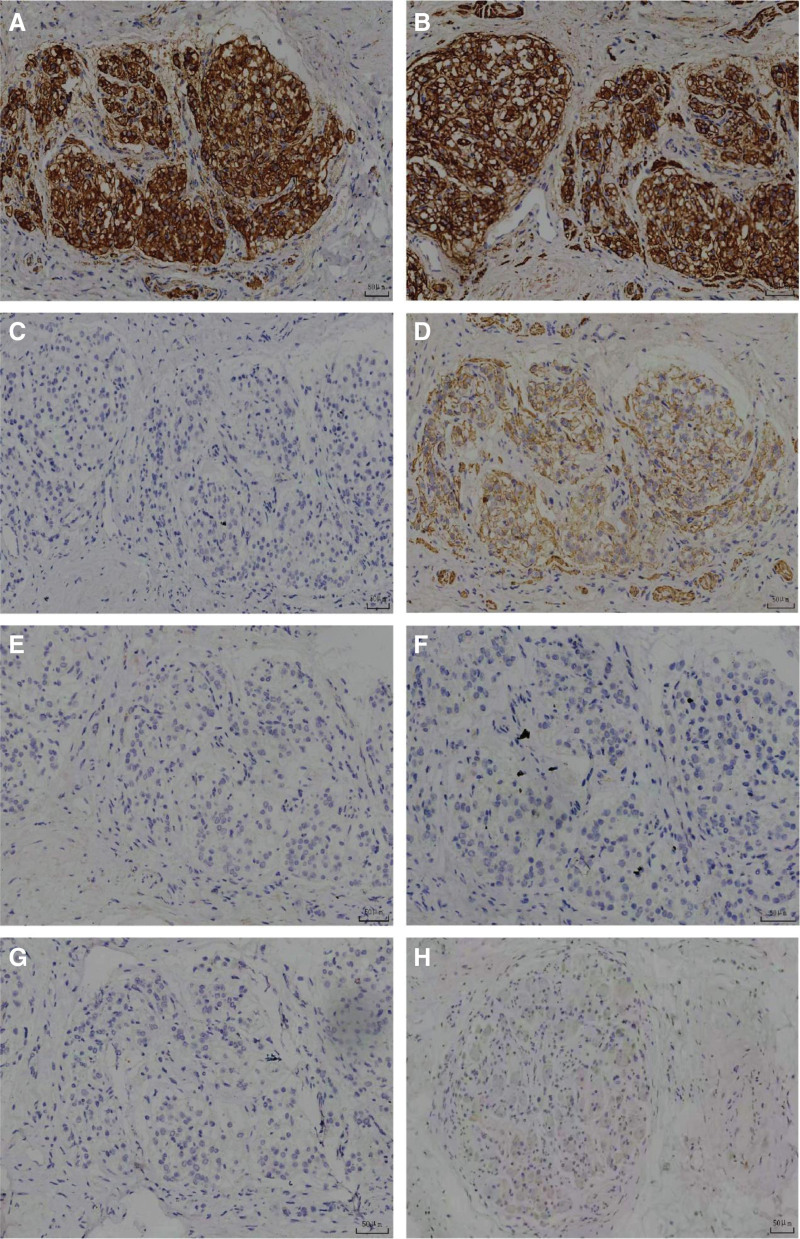
Immunohistochemical results. (A) Syn (+), (B) Melan-A (+), (C) CD56 (-), (D) α-inhibin (+), (E) PSA (-), (F) P504S (-), (G) CgA (-), (H) EMA (-).

Unexpectedly, postoperative pathology also revealed the presence of adrenal tissues around the prostate (the adrenal tissue was located outside the prostatic capsule), arranged in a nest-like distribution. The ectopic adrenal tissue was circular in shape and included 5 lesions located around the prostate. The largest lesion diameter was 787.75 μm (lesion 2), and the smallest was 242.11 μm (lesion 3). High-magnification examination showed that the adrenal tissue was composed of adrenal cortex. The cell and nucleus are large, and the cytoplasm contains a large number of lipid droplets in a foamy shape (Fig. 2A–C). Immunohistochemistry revealed Syn (+), Melan-A (+), CD56 (-), α-inhibitor (+), PSA (-), P504S (-), CgA (-), EMA (-) (Fig. 3A–H). Therefore, the diagnosis of ectopic adrenal tissue around the prostate was confirmed.

**Figure 4.**
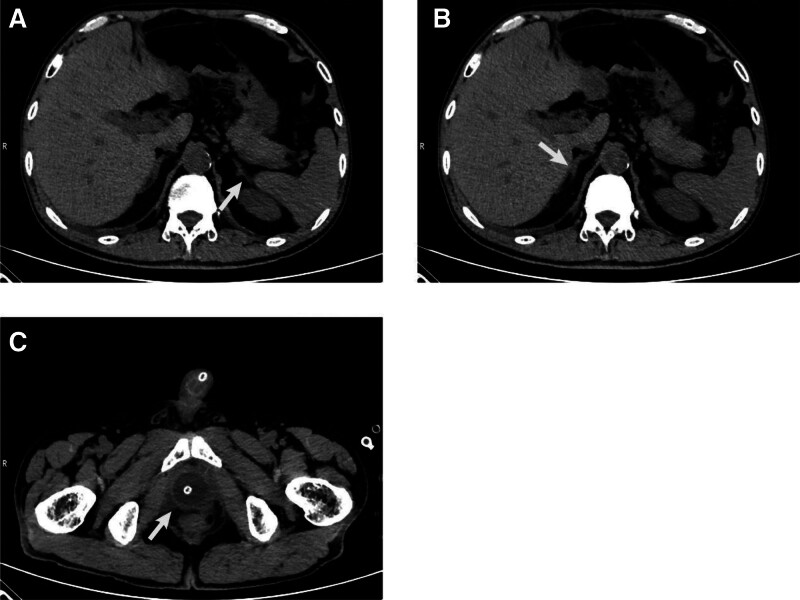
CT of the patient 1 month after surgery. No significant abnormalities were found in the left (A) and right (B) adrenal glands. (C) No significant abnormalities were observed after radical cystectomy.

The patient was discharged 9 days after surgery and no significant abnormalities were found during follow-up (Fig. 4A–C).

## 4. Discussion

Ectopic adrenal tissue is rare, typically discovered incidentally by surgeons or pathologists.^[3]^ It is more commonly identified in children, as ectopic adrenal tissue tends to atrophy or disappear in adults.^[4]^ Therefore, the incidence of ectopic adrenal tissue in adults is <1%.^[5]^

Currently, ectopic adrenal tissue has been reported in the region around the adrenal gland, the celiac plexus, the kidney, and the route of gonadal descent, comprising also hernia sacs.^[5–9]^ Additionally, it also can be found in bizarre sites, such as placenta,^[10]^ lung, etc (Table S1, Supplemental Digital Content, https://links.lww.com/MD/Q789).^[11,12]^ However, to our knowledge, ectopic adrenal tissue around the prostate is extremely rarely reported.

The etiology of adrenal tissue ectopia remains controversial.^[3]^ Most scholars believe that ectopic adrenal tissue may originate either from multiple primordia or from secondarily detached cortical fragments during the penetration of medullary cells into cortical anlage.^[1,4]^ Subsequently, these tissues may remain close to the main gland or migrate, mostly in the pelvis or the groin region.^[3]^ In addition, some scholars have also proposed the pluripotent cells theory to explain the atypical locations of adrenal ectopia.^[1]^ In this patient, the 1st theory seems plausible for explaining ectopic adrenal tissue around the prostate, as the adrenal glands and the prostate both originate from the mesoderm. However, this speculation needs further confirmation due to the lack of long-term tracking of embryonic development.

Most ectopic adrenal tissues are asymptomatic and incidentally discovered.^[3]^ However, limited reports suggest that ectopic adrenal tissue exhibits hormonal activity and responds to hormone stimulation such as adrenocorticotrophin.^[13,14]^ When ectopic adrenal tissue is removed, adrenal insufficiency may occur (This situation only occurs when the adrenal gland located in the renal parenchyma is accidentally removed during nephrectomy, or when the removed adrenal tissue is overactive and suppresses the normal function of the adrenal gland).^[5]^ Conversely, when the normal adrenal glands are removed, ectopic adrenal tissue can proliferate.^[15]^ This suggests that ectopic adrenal tissue possesses hormone-secreting capabilities. If ectopic adrenal tissue exhibits strong hormone-secreting capabilities, it can lead to hormonal metabolic disturbances.^[1]^ Furthermore, tumors can develop by neoplastic transformation of ectopic tissue with or without hormone production.^[3,16,17]^ When these problems arise, the diagnosis and treatment may be difficult for clinicians. In this patient, adrenal hormone testing was not performed due to the incidental discovery of ectopic adrenal tissue without any symptoms of adrenal hormone abnormalities. However, we speculate that the ectopic adrenal tissue is likely nonfunctional, as there were no symptoms related to hormonal changes observed postoperatively, and CT revealed no significant abnormalities in the adrenal glands (Fig. 4A, B).

Macroscopically, ectopic adrenal tissue typically appears as a round, yellow, well-defined nodule, usually smaller than 1.0 cm.^[3]^ Microscopically, it generally contains only cortical components, although some cases, especially those located in the celiac plexus, have shown both cortical and medullary components.^[5,13]^ In this patient, the ectopic adrenal tissue was not observable macroscopically but was clearly visible under the microscope, containing only adrenal cortex.

## 5. Conclusion

This case represents the ectopic adrenal tissue around the prostate, broadening our understanding of adrenal tissue ectopia. Ectopic adrenal tissue around the prostate contains only the cortex and appears to be nonfunctional. Due to its small size and rarity, detecting ectopic adrenal tissue in the prostate through imaging is challenging.

## Author contributions

**Supervision**: Changxing Ke.

**Writing – original draft**: Anlian Wang, Tao Zhang, Yingxiong Yang, Guorun Zi.

**Writing – review & editing**: Jiagui Chai, Runlin Feng, Yuhang Li.

## Supplementary Material

## References

[1] Schechter DC. Aberrant adrenal tissue. Ann Surg. 1968;167:421–6. 5638527 10.1097/00000658-196803000-00017 PMC1387073

[2] Okur H Küçükaydin M Kazez A Kontaş O. Ectopic adrenal tissue in the inguinal region in children. Pediatr Pathol Lab Med. 1995;15:763–7. 8597861 10.3109/15513819509027011

[3] Falco EC Daniele L Metovic J. Adrenal rests in the urogenital tract of an adult population. Endocr Pathol. 2021;32:375–84. 34095993 10.1007/s12022-021-09685-y PMC8370964

[4] Anderson JR Ross AH. Ectopic adrenal tissue in adults. Postgrad Med J. 1980;56:806–8. 7267489 10.1136/pgmj.56.661.806 PMC2426068

[5] Senescende L Bitolog PL Auberger E Zarzavadjian Le Bian A Cesaretti M. Adrenal ectopy of adult groin region: a systematic review of an unexpected anatomopathologic diagnosis. Hernia. 2016;20:879–85. 27601037 10.1007/s10029-016-1535-1

[6] Gutowski WT 3rd Gray GF Jr. Ectopic adrenal in inguinal hernia sacs. J Urol. 1979;121:353–4. 430634 10.1016/s0022-5347(17)56783-x

[7] El Demellawy D Nasr A Samkari A Pastolero P Alowami S. Aberrant adrenocortical tissue in hernia sac occurring in an adult: case report and review of the literature. Hernia. 2009;13:659–62. 19367442 10.1007/s10029-009-0501-6

[8] Iyengar V Pittman DM. Ectopic adrenal gland tissue in inguinal hernia sac. Ann Diagn Pathol. 2007;11:291–2. 17630115 10.1016/j.anndiagpath.2006.08.001

[9] Mendez R Tellado MG Somoza I. Ectopic adrenal tissue in the spermatic cord in pediatric patients: surgical implications. Int Braz J Urol. 2006;32:202–7; discussion 207. 16650300 10.1590/s1677-55382006000200013

[10] Labarrere CA Caccamo D Telenta M Althabe O Gutman R. A nodule of adrenocortical tissue within a human placenta: light microscopic and immunocytochemical findings. Placenta. 1984;5:139–43. 6237324 10.1016/s0143-4004(84)80057-0

[11] Armin A Castelli M. Congenital adrenal tissue in the lung with adrenal cytomegaly. Case report and review of the literature. Am J Clin Pathol. 1984;82:225–8. 6465087 10.1093/ajcp/82.2.225

[12] Wiener MF Dallgaard SA. Intracranial adrenal gland; a case report. AMA Arch Pathol. 1959;67:228–33. 13616833

[13] Falls JL. Accessory adrenal cortex in the broad ligament: incidence and functional significance. Cancer. 1955;8:143–50. 13231045 10.1002/1097-0142(1955)8:1<143::aid-cncr2820080120>3.0.co;2-p

[14] Mori H Matsumoto K. Constant occurrence of adrenocortical tissue in the juvenile rabbit ovary. Am J Anat. 1974;141:73–90. 4368535 10.1002/aja.1001410105

[15] Johnson DN Sadow PM. Exploration of BRAFV600E as a diagnostic adjuvant in the non-invasive follicular thyroid neoplasm with papillary-like nuclear features (NIFTP). Hum Pathol. 2018;82:32–8. 30146440 10.1016/j.humpath.2018.06.033

[16] van Ingen G Schoemaker J Baak JP. A testosterone-producing tumour in the mesovarium. Pathol Res Pract. 1991;187:362–70; discussion 370. 2068021 10.1016/S0344-0338(11)80807-1

[17] Jain SH Sadow PM Nosé V Dluhy RG. A patient with ectopic cortisol production derived from malignant testicular masses. Nat Clin Pract Endocrinol Metab. 2008;4:695–700. 18941436 10.1038/ncpendmet0985

