# Supplementary material for: Ectopic adrenal tissue around the prostate: A rare case
Source: Medicine (Baltimore). 2026 May 12;104(49):e46287. doi: 10.1097/MD.0000000000046287 (PMC12688945; doi:10.1097/MD.0000000000046287)
Supplement: Supplementary file 1 [file medi-104-e46287-s001.docx]

**Supplementary Table 1** Main reports on adrenal ectopia.

| Ectopic location | Age | Gender | How to discover | Symptom | Adrenal function | Treatment | References |
| --- | --- | --- | --- | --- | --- | --- | --- |
| Lung | 5 days old | male | Accidentally discovered through autopsy | Asymptomatic | - | - | [1] |
| Inguinal ligament | 34 years old | male | Accidentally discovered after surgery | Asymptomatic | Not examined | None | [2] |
| Intratesticular | 34 years old | male | Accidentally discovered after surgery | Asymptomatic | Not examined | None | [2] |
| Spermatic cord | 31 years old | male | Accidentally discovered after surgery | Asymptomatic | Not examined | None | [3] |
| Hernia sac | 78 years old | male | Accidentally discovered after surgery | Asymptomatic | Not examined | None | [4] |
| ovary | 68 years old | female | Accidentally discovered after surgery | Asymptomatic | Not examined | None | [5] |
| liver | 49 years old | female | Accidentally discovered after surgery | Asymptomatic | Not examined | None | [6] |
| placenta | 26 years old | female | Accidentally discovered after surgery | Asymptomatic | Not examined | None | [7] |
| renal | 35 years old | female | Accidentally discovered after surgery | Asymptomatic | Not examined | None | [8] |

**References**

[1] Armin A, Castelli M. Congenital adrenal tissue in the lung with adrenal cytomegaly: Case report and review of the literature. American journal of clinical pathology. 1984;82:225-8.

[2] Czaplicki M, Bablok L, Kuzaka B, et al. Heterotopic adrenal tissue. International urology and nephrology. 1985;17:177-81.

[3] Gualtieri T, Segal AD. Report of a case of adrenal-type tumor of the spermatic cord; a review of aberrant adrenal tissues. The Journal of urology. 1949;61:949-55.

[4] El Demellawy D, Nasr A, Samkari A, et al. Aberrant adrenocortical tissue in hernia sac occurring in an adult: case report and review of the literature. Hernia : the journal of hernias and abdominal wall surgery. 2009;13:659-62.

[5] Lee SM, Baek JC, Park JE, et al. Ectopic adrenal gland tissue in the left ovary of an elderly woman: a case report. The Pan African medical journal. 2021;40:181.

[6] Chai J, Feng R, Ke C. Ectopic adrenal tissue in the liver: A rare case. Asian journal of surgery. 2024; 24:S1015-9584.

[7] Labarrere CA, Caccamo D, Telenta M, et al. A nodule of adrenocortical tissue within a human placenta: light microscopic and immunocytochemical findings. Placenta. 1984;5:139-43.

[8] Ye H, Yoon GS, Epstein JI. Intrarenal ectopic adrenal tissue and renal-adrenal fusion: a report of nine cases. Modern pathology : an official journal of the United States and Canadian Academy of Pathology, Inc. 2009;22:175-81.
